# Combination of partial least square structural equation modeling scheme of principal component analysis with importance performance analysis^☆^^☆☆^

**DOI:** 10.1016/j.mex.2025.103783

**Published:** 2025-12-30

**Authors:** Bambang Widjanarko Otok, Zulfani Alfasanah, Diaz Fitra Aksioma

**Affiliations:** Departement of Statistic, Faculty of Science and Data Analytics (Scientics), Institut Teknologi Sepuluh Nopember, Surabaya, Indonesia

**Keywords:** Importance-performance analysis, Partial least square, Principal component analysis, Structural Equation Modeling

## Abstract

Structural Equation Modeling (SEM) is widely used to assess causal relationships among latent variables, yet its strict assumptions often limit empirical applications. Partial Least Squares SEM (PLS-SEM) offers greater flexibility, but the choice of weighting scheme remains a methodological challenge. This study introduces a PCA-based weighting scheme to improve the stability and accuracy of PLS estimation. Importance-Performance Analysis (IPA) is further integrated to identify high-impact but underperforming indicators. Applied to child malnutrition in East Java, the approach reveals that socio-economic conditions most strongly influence food security, parenting, and health–environment services. IPA highlights exclusive breastfeeding as a priority for intervention. The proposed methodological approach strengthens PLS estimation and yields actionable insights for prioritizing policy measures.

## Highlights

• PCA is used in the inner weighting scheme of the PLS model to obtain latent variable scores.
• PLS-IPA explains the influence between latent variables and indicators while mapping indicators based on importance and performance.

## Graphical abstract

Graphical abstract for Combination of Partial Least Square Structural Equation Modeling Scheme of Principal Component Analysis with Importance Performance Analysis.

**Figure:**
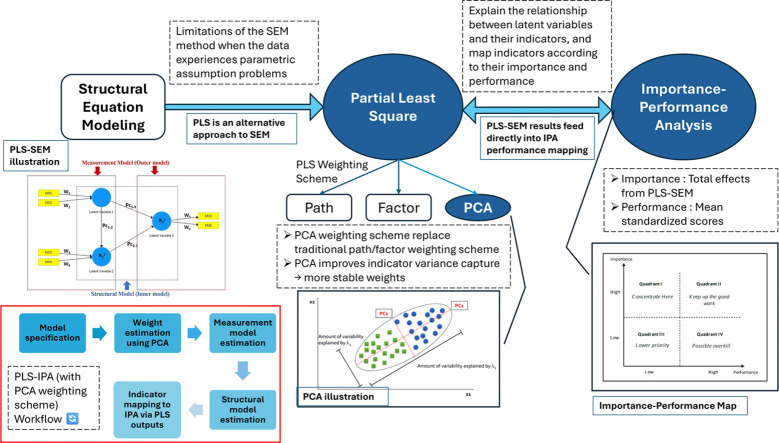

**Specifications table**

**Table:** 

**Subject area**	Mathematics and Statistics
**More specific subject area**	Statistics; Multivariat Data Analysis; Structural Equation Modeling
**Name of your method**	Partial Least Square – Importance Performance Analysis
**Name and reference of original method**	B. W. Otok, Purhadi, R. Sriningsih, and D. S. Dila, “Segmentation of toddler nutritional status using REBUS and FIMIX partial least square in Southeast Sulawesi,” *MethodsX*, vol. 12, p. 102515, 2024, doi: 10.1016/j.mex.2023.102515.
**Resource availability**	SmartPLS, RStudio

## Background

Structural Equation Modeling is a multivariate statistical method that integrates regression analysis, factor analysis, and path analysis to model complex relationships between variables. Despite its widespread use, conventional SEM has methodological limitations, particularly the assumptions of multivariate normality, independence of observations, and the need for relatively large sample sizes. In empirical research, data often do not meet these requirements, especially when sample sizes are limited or data distributions are non-normal [1].

As an alternative, Partial Least Squares Structural Equation Modeling was developed to address these limitations. PLS-SEM operates without the assumption of a normal distribution, can be used on various data scales, works with small sample sizes, and is suitable for exploratory research and initial theory development. Based on a composite approach, PLS-SEM allows for the estimation of causal relationships between latent variables, even when the model being analyzed is complex [2].

In the PLS-SEM algorithm, there are three commonly used weighting schemes: path, centroid, and factor. Each scheme has distinct characteristics, including causal orientation, sensitivity to correlations between latent variables, and requirements for specific data conditions [3]. This study integrates Principal Component Analysis into PLS-SEM as a scheme for selecting inner model weights. PCA reduces data dimensionality by extracting principal components, thus making the weight estimation process more structured and focused on components with the greatest contribution to data variance. This method aims to transform a set of correlated variables into a new set of uncorrelated variables, referred to as principal components [4].

Furthermore, various PLS-SEM extensions have been developed to expand the analysis capabilities. Previous studies still tend to use standard PLS path modeling, while advanced approaches such as FIMIX-PLS, PLS-POS, CTA-PLS, moderator analysis, and multigroup analysis have not been widely utilized [5]. One development in this research is Partial Least Squares Importance-Performance Analysis, which combines the dimensions of importance and performance to map construct and indicator priorities. IPA is an approach used to evaluate the quality of a system based on its level of importance and performance achievement. IPA is flexible because it can be used on various types of data, both qualitative and quantitative, and can be integrated with other analysis techniques to strengthen interpretations [6].

Several previous studies support this research. Hock et al. (2010) used PLS and IPA to evaluate visitor satisfaction factors at a multifunctional stadium [7]. Hauff et al. (2024) combined IPA, PLS, and Necessary Condition Analysis in an integrative analytical framework [8]. In the health sector, Lee et al. (2021) applied IPA to examine adolescents' health perceptions during the COVID-19 pandemic [9], Lefrandt et al. (2016) combined SEM and IPA to evaluate pedestrian satisfaction on pedestrian paths in Manado City [10]. In health and environmental contexts, IPA has also been used to identify priority intervention factors and examine the distribution of resource [6].

However, studies specifically combining the PLS-SEM PCA scheme with IPA are still limited. Therefore, this study aims to fill this gap by examining the application of the combined PLS-SEM PCA scheme with IPA in a relevant empirical context.

## Method details

### Partial least squares structural equation modeling

PLS-SEM is a flexible technique for analyzing latent variables represented by multiple observed indicators. Unlike covariance-based SEM, which relies on a covariance-oriented framework, PLS-SEM employs a variance-based approach. This makes it particularly advantageous when dealing with data limitations such as small sample sizes, categorical or non-interval scales, missing values, non-normal distributions, or multicollinearity. One of its main benefits is its ability to handle all types of measurement scales (nominal, ordinal, interval, and ratio) with relatively relaxed statistical assumptions. Unlike covariance-based SEM, PLS-SEM does not require strict conditions such as multivariate normality, rigid measurement specifications, or large sample sizes. This makes it highly effective for addressing multicollinearity and especially suitable for exploratory research [2].

The structural model specifies theoretical relationships among latent variables, can be written as Eq. (1).

(1) η=βη+Γξ+ζ

where **η** and **ξ** represent endogenous and exogenous latent variables, respectively. Path coefficients in matrices **B** and **Γ** quantify direct and indirect effects between latent variables. The measurement model defines how latent variables relate to their observed indicators, either reflectively or formatively [11].

In reflective models, indicators mirror their latent variable, as represented by Eqs. (2) and (3).

(2) $$y_{\left(p\times 1\right)}=\Lambda_{y(p\times m)}\eta_{\left(m\times 1\right)}+{ɛ}_{\left(p\times 1\right)}$$

(3) $$x_{\left(q\times 1\right)}=\Lambda_{x(p\times n)}\xi_{\left(n\times 1\right)}+\delta_{\left(q\times 1\right)}$$

In these equations, $\Lambda_{y}$ and $\Lambda_{x}$ represent the loading matrices connecting latent variables with their indicators, while ɛ and δ are the measurement error terms for endogenous and exogenous indicators, respectively. The parameters p and q indicate the number of indicators for endogenous and exogenous latent variables, whereas m and n denote the number of latent variables [11].

Since both structural and measurement models are defined conceptually, the scores of latent variables are not directly observed. To obtain these scores, outer weights must be estimated. A major strength of PLS-SEM is that it allows latent variable scores to be computed as linear combinations of their observed indicators. These scores are derived using iterative weight estimation procedures in the PLS algorithm, as represented in Eqs. (4) and (5), where $w_{jh}$ and $w_{ih}$ are the weights used to estimate the latent variables $\xi_{j}$ and $\eta_{i}$, respectively [12].

(4) $${\hat{\xi }}_{j}=\sum_{h=1}^{H}w_{jh}x_{jh}$$

(5) $${\hat{\eta }}_{i}=\sum_{i=1}^{I}w_{ih}y_{ih}$$

The main aim of PLS-SEM is to predict and explain the variance in endogenous latent variables by estimating a system of hypothesized relationships, similar to the principle used in Ordinary Least Squares (OLS) regression [13]. Parameter estimation in PLS-SEM proceeds through a two-stage algorithm. In the first stage, the model iteratively estimates outer weights and latent variable scores. It starts with an outside approximation—computing latent scores as linear combinations of their indicators—then updates these scores through inside approximation using inner weights derived from one of three schemes: path, centroid, or factor weighting. The process repeats until convergence, indicated when the change in outer weights between iterations falls below a set threshold (typically 10⁻⁵). Once convergence is achieved, the final latent variable scores are used to estimate the relationships among latent variables. The second stage applies Ordinary Least Squares regression to obtain path coefficients for the structural model and loadings for the measurement model, thus allowing simultaneous evaluation of both models without strict data assumptions [14]

• Bootstrap Method in PLS

Since PLS-SEM does not require the data to follow a multivariate normal distribution, conventional parametric significance tests for path coefficients, loadings, and outer weights cannot be applied. Instead, PLS utilizes a nonparametric resampling technique to assess the significance of its model parameters [13]. The bootstrap method, first proposed by Efron (1979), is a nonparametric inference approach designed to overcome issues tied to normality assumptions. It serves to estimate parameters of an unknown population distribution, measure sampling variability, and calculate standard errors. In general, the approach is carried out on a data sample $x=\{x_{1},x_{2},...,x_{n}\}$ from a population with an unknown distribution function, namely *F*, to estimate the parameter θ=t(F) [15].

Within the PLS context, bootstrapping entails taking samples with replacement from the original dataset to produce pseudo-samples that preserve the characteristics of the original data. This repeated resampling allows for the calculation of standard errors, which are then used to evaluate the reliability and significance of estimates in both the measurement and structural models. The bootstrap standard error for an estimator θ ^ is defined as the standard deviation of the B bootstrap replications, as shown in Eq. (6).

(6) $$se\left({\hat{\theta }}^{*}\right)=\sqrt{\frac{\sum_{b=1}^{B}\left({\hat{\theta }}_{\left(b\right)}^{*}-{\hat{\theta }}_{\left(.\right)}^{*}\right)}{B-1}}\;\text{with}\;{\hat{\theta }}_{\left(.\right)}^{*}=\frac{\sum_{b=1}^{B}{\hat{\theta }}_{\left(b\right)}^{*}}{B}$$

where $\sum_{b=1}^{B}\left({\hat{\theta }}_{\left(b\right)}^{*}-{\hat{\theta }}_{\left(.\right)}^{*}\right)$ is the number of resampling sets of size n with replacement, and ${\hat{\theta }}_{\left(b\right)}^{*}$ is the original data statistic $\hat{\theta }$ calculated from repeated samples b (b=1,2,3,...,B). $se({\hat{\theta }}^{*})$ is defined as a plug-in estimate of $\hat{F}$ as a substitute for the unknown distribution of *F* and is defined as $se_{\hat{F}}\left({\hat{\theta }}^{*}\right)$ [16].

The procedure for estimating standard errors using the bootstrap method involves several steps carried out sequentially. First, *B* independent bootstrap samples of size *n* are generated by drawing observations with replacement from the original dataset *X*. Next, the model parameters are estimated for each of these *B* bootstrap samples, resulting in *B* replications of the parameter estimates. Finally, the standard error for each parameter is calculated based on the variability observed across these *B* replications, following the formula described earlier [15].

In the measurement model, bootstrapping makes it possible to test whether factor loadings significantly differ from zero. The bootstrap-derived standard errors are then used to perform a *t*-test, as expressed in Eq. (7), where $\hat{\lambda_{i}}$ is the estimated value of $\lambda_{i}$ and $se(\hat{\lambda_{i}})$ is the standard error for $\hat{\lambda_{i}}$ [13].

(7) $$T=\frac{\hat{\lambda_{i}}}{se\left(\hat{\lambda_{i}}\right)}$$

Likewise, in the structural model, the same principle applies for testing hypotheses related to path coefficients (β and γ). Their t-statistics are calculated following Eqs. (8) and (9). As the name suggests, the test statistic follows a t-distribution with degrees of freedom (df) of the number of observations minus 1 (*n* – 1). The t-distribution is approximated by a normal (Gaussian) distribution for observations greater than 30. In bootstrapping, the number of observations often exceeds the threshold (>1000 bootstrap samples). Therefore, when the *t*-test statistic value exceeds the critical limit of 1.96, it can be assumed that the loading factor or path coefficient value is significantly different from zero at the 5 % significance level (α=0,05; two-tailed test) [13].

(8) $$T=\frac{\hat{\beta_{i}}}{se\left(\hat{\beta_{i}}\right)}$$

(9) $$T=\frac{\hat{\gamma_{i}}}{se\left(\hat{\gamma_{i}}\right)}$$

• PLS Model Evaluation

Model evaluation in PLS is conducted using a nonparametric, predictive approach. The evaluation process includes assessing both the measurement model and the structural model [17].

a) Structural Model

Evaluation of the measurement model with reflective indicators consists of validity and reliability.

1. Validity

A latent variable is declared valid if it meets convergent and discriminant validity. Convergent validity indicates the extent to which indicators reflecting a latent variable are positively correlated. This is evaluated through factor loadings (ideal > 0.7, tolerance > 0.5) and Average Variance Extracted (AVE > 0.5), indicating that the indicator is able to explain at least 50 % of the latent variable's variance. The calculation refers to Eq. (10), where ${\hat{\lambda }}_{i}$ indicates the i^th^ component loading of the indicator and $\text{var}\left(\varepsilon_{i}\right)=1-{\hat{\lambda }}_{i}^{2}$ [18].

(10) $$AVE=\frac{\sum_{i=1}^{I}{\hat{\lambda }}_{i}^{2}}{\sum_{i=1}^{I}{\hat{\lambda }}_{i}^{2}+\sum_{i=1}^{I}\text{var}\left(\varepsilon_{i}\right)}$$

Discriminant validity ensures that indicators can differentiate one latent variable from another. Evaluation is performed by examining cross-loadings (an indicator must be higher on its own latent variable than on other latent variables) and the Fornell-Larcker Criterion (the square root of a construct's AVE must be greater than its correlation with other latent variables) [18].

2. Reliability

Reliability is used to assess the internal consistency of a latent variable. Two commonly used measures are Cronbach's alpha and composite reliability, with composite reliability considered more accurate. Cronbach's alpha tends to provide a lower limit value because it assumes all indicators have the same weight (or equivalence), while composite reliability does not make this assumption and is therefore more accurate in measuring latent variable reliability. This method only applies to latent variables with reflective indicators. Composite reliability values range from 0 to 1, and a latent variable is considered reliable if its value is above 0.7. Composite reliability is formulated in Eq. (11), where ${\hat{\lambda }}_{i}$ indicates the i^th^ component loading of the indicator and $\text{var}\left(\varepsilon_{i}\right)=1-{\hat{\lambda }}_{i}^{2}$ [19].

(11) $$\rho_{c}=\frac{{\left(\sum_{i=1}^{I}{\hat{\lambda }}_{i}\right)}^{2}}{{\left(\sum_{i=1}^{I}{\hat{\lambda }}_{i}\right)}^{2}+\sum_{i=1}^{I}\text{var}\left(\varepsilon_{i}\right)}$$

a) Measurement Model

1. Coefficient of Determination

One of the statistical indicators in evaluating a structural model is the coefficient of determination (R²). This measure indicates the proportion of variation in the endogenous latent variable that can be explained by the exogenous latent variable. An R² value of 0.19 indicates a weak influence, 0.33 indicates a moderate influence, and 0.67 reflects a strong influence. The R² calculation can be done as shown in Eq. (12) [19].

(12) $$R^{2}=\sum_{h=1}^{H}{\hat{\beta }}_{jh}corr\left(X_{jh},Y_{j}\right)$$

2. Goodness of Fit Index

Goodness of Fit (GoF) is a single measure used to assess the overall performance of a model, namely a combination of the measurement model and the structural model. The GoF value is calculated from the multiplication of the average AVE by the average R², as shown in Eq. (13) [20].

(13) $$GoF=\sqrt{\bar{AVE}\times \bar{R^{2}}}$$

3. Q^2^ Predictive Relevance

Q^2^ predictive relevance is used to test the predictive ability of the model. If the Q² value is close to 1, then the structural model is considered to fit the data and has good predictive relevance. The calculation formula is shown in Eq. (14) [20].

(14) $$Q^{2}=1-\left(1-R_{1}^{2}\right)\left(1-R_{2}^{2}\right)...\left(1-R_{j}^{2}\right)$$

### Principal component analysis

PCA is a multivariate statistical technique designed to reduce the dimensionality of a dataset while preserving its most essential information. PCA transforms a set of correlated observed variables into a new set of orthogonal and uncorrelated components, known as principal components, which are linear combinations of the original indicators. These components are ordered according to the amount of variance explained, with the first principal component capturing the maximum possible variance in the data [4]. Within the PLS-SEM framework, PCA serves as one of the available weighting schemes, alongside the factor and path weighting schemes. Integrating PCA into the PLS-SEM algorithm enhances analytical efficiency by reducing indicator dimensionality and producing uncorrelated composite scores. The primary advantage of the PCA weighting scheme lies in its ability to extract the maximum variance from indicators into the latent variable scores. In contrast to the path and centroid schemes, which depend on structural model relationships, the PCA scheme prioritizes variance maximization through the first principal component, enabling more efficient and information-rich latent variable estimation [21].

Mathematically, PCA in the context of PLS-SEM seeks linear combinations of indicator variables to form latent scores. The latent score t can be obtained through a linear transformation:

t=Xw

where X is the indicator data matrix and w is the weight vector chosen so that the variance of t is maximized:

$$w=\arg\;\max_{w}\;Var\left(\mathrm{Xw}\right)$$

with the normalization constraint:

∥w∥=1

In PCA implementation, the weight vector w is obtained as an eigenvector of the covariance matrix $X^{T}X$:

$$X^{T}\mathrm{Xw}=\lambda w$$

where λ is an eigenvalue indicating the amount of variance explained by that principal component. The proportion of variance represented by each eigenvector can be calculated by dividing the eigenvalue corresponding to that eigenvector by the sum of all eigenvalues. The first weight obtained from the eigenvector with the largest eigenvalue will be used to form the first latent score, which is then used in the inner regression of the PLS-SEM model. This approach ensures that the formed components capture maximum information from the indicator variables while eliminating redundancy due to correlation between indicators [21].

The following schematic outlines the sequential stages of the PLS algorithm while explicitly illustrating how Principal Component Analysis (PCA) is incorporated into the initialization and refinement of the weighting scheme.

**Step 1–8: Preprocessing and Structural Mapping**

1. Initialize an empty list structure for storing outputs.
2. Identify the total number of indicators.
3. Identify the total number of latent variables.
4. Identify the number of observations.
5. Standardize the raw dataset to generate a standardized data matrix.
6. Map each indicator to its corresponding latent variable.
7. Specify the type of outer measurement model (reflective or formative).
8. Convert standardized matrices into list formats for model computation.

**Step 9: PCA-Based Weight Extraction**

For each latent variable:

• Extract the associated standardized indicator block.
• Perform Principal Component Analysis (PCA) on the block.
• Extract the loading vector of the first principal component (PC1).
• Normalize the loading vector such that the sum of absolute values equals one.
• Treat this normalized vector as the final weight vector for the latent variable.

**Step 10–12: Latent Variable Score Construction and Outer Weight Calculation**

10. Compute the latent variable score for each latent variable.

11. Compute outer weights (reflective or formative) using the PCA-based latent variable score.

12. Apply outside approximation as required by the measurement model.

**Step 13–16: Structural and Measurement Model Estimation**

13. Identify exogenous latent variables.

14. Assemble a matrix of exogenous latent variable scores.

15. Estimate path coefficients using ordinary least squares (OLS).

16. Compute outer loadings.

**Step 17: Estimation of Location Parameters**

17. Compute the location parameters m, nx, $p_{0}$, and $b_{0}$ to adjust the latent variable score positions.

These corrections ensure comparability across latent variables and align the results with PLS-IPA requirements.

The application of the PCA scheme in PLS-SEM offers several significant advantages over conventional weighting schemes. First, PCA can handle multicollinearity issues by transforming correlated variables into orthogonal components. Second, the focus on variance maximization allows for optimal information extraction from the dataset. Third, the numerical stability achieved by PCA provides more consistent convergence in the PLS-SEM algorithm, especially in situations with high data complexity [22].

### Importance-Performance analysis

The Importance-Performance Analysis (IPA) method is an approach to assessing service quality based on the level of importance and customer satisfaction. IPA analysis uses a quadrant diagram with two main dimensions: importance on the vertical axis and satisfaction on the horizontal axis. The intersection of the two axes is determined by the average importance and satisfaction values. Through this mapping, service providers can identify attributes that need to be maintained or improved to optimize customer satisfaction [10].

**Fig. 1:**
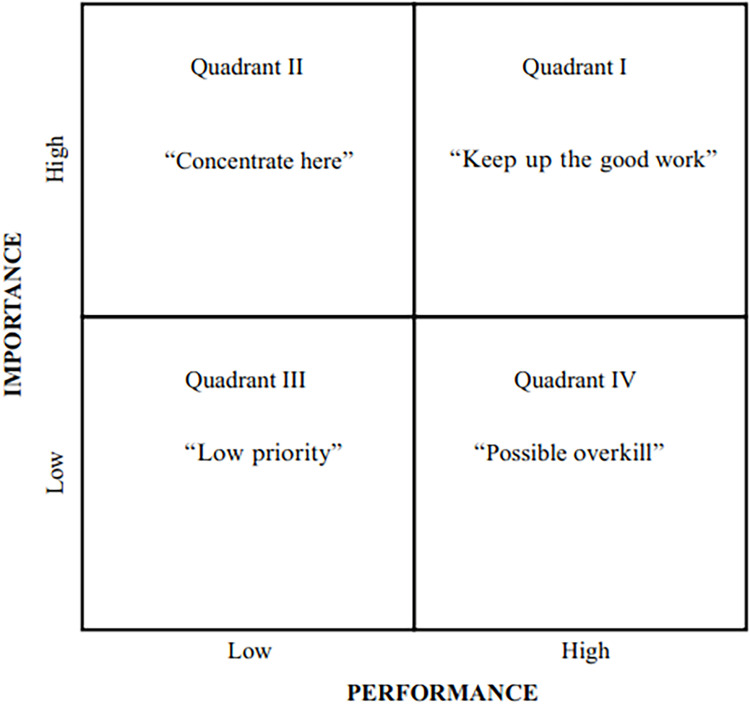
IPA Base Map.

IPA has been widely applied in various fields, such as marketing and management, and customer satisfaction studies, as it helps organizations set strategic priorities. In IPA, factors are mapped into four quadrants as seen in Fig. 1. The first quadrant (Keep Up the Good Work) includes factors with high importance and performance, which should be maintained as organizational strengths. The second quadrant (Concentrate Here) contains important factors with low performance, and therefore should be prioritized for improvement. The third quadrant (Low Priority) contains factors with low importance and performance, which do not require significant attention unless their relevance increases in the future. Meanwhile, the fourth quadrant (Possible Overkill) indicates factors with high performance but low importance, and therefore resource allocation for this aspect should be reviewed [23].

### PLS-IPA

Partial Least Squares is a composite-based structural equation modeling approach in which a latent variable is represented as a weighted combination of its indicators [14]. This method allows the calculation of a composite score, often interpreted as a performance value, representing respondents' assessments of a particular attribute or latent variable. For example, if respondents indicate maximum satisfaction with a latent variable, this is reflected in a performance score of 100 % [24]. The basic characteristics of PLS-SEM were then utilized in the development of standardized performance indices. Based on this principle, subsequent studies combined performance scores from PLS-SEM with importance measures derived from weights in structural and measurement models. The resulting integration gave rise to the Partial Least Squares-Importance Performance Analysis (PLS-IPA) method. In PLS-IPA, importance scores are plotted on the horizontal axis, while performance scores are displayed on the vertical axis. This mapping visualizes the strategic position of the latent variable relative to the intended outcome variable. To assess importance, PLS-IPA generally uses total effects in structural models, which include both direct and indirect influences between latent variables. This method provides a comprehensive overview of the extent to which antecedent latent variables influence the target outcome. Latent variables with high total effects but low performance are prioritized for improvement, as their improvement has the potential to have a significant impact [8,25].

The performance aspect itself is represented by the average score for each latent variable. This score can be calculated using either standardized or unstandardized data. However, standardized scores (z-transformed) are less useful because they always have a mean of zero and a standard deviation of one. Conversely, unstandardized scores are preferred because they remain on the original measurement scale, making them easier to interpret in practice. Challenges arise when the scales between indicators differ; for this, rescaling is performed to a range of 0–100, where 0 indicates the lowest performance and 100 the highest performance (Eq. (15)). This rescaling can use theoretical or empirical minimum and maximum values, so that results between latent variables can be compared equally [26].

(15) $$x_{\text{ij}}^{\text{rescaled}}=\frac{E\left[x_{\text{ij}}\right]-\text{min}\left[x_{i}\right]}{\max\left[x_{i}\right]-\text{min}\left[x_{i}\right]}.100$$

Scaled latent variable scores are calculated as a linear combination of rescaled indicators with adjusted external weights. These weights are obtained by transforming standardized weights into unstandardized weights, then normalizing them to total one in each measurement model. The final result is a performance score ranging from 0 to 100, which is then combined with the importance score to construct an importance-performance map [26].

The importance values are obtained from the total effects of the indicators on the target latent variable, which are calculated by multiplying the rescaled outer weights of the indicators belonging to a predecessor latent variable with the unstandardized total effect of that latent variable on the target latent variable. In this sense, importance reflects the extent to which an indicator, through its associated latent variable, contributes to explaining the target latent variable. Conversely, the performance values are derived from the mean values of the rescaled data of each indicator, thereby representing the actual level of achievement of the indicators on a standardized scale. By integrating both importance and performance values across all indicators, an importance-performance map can be constructed. This analytical tool provides a systematic framework for identifying indicators that exert a substantial influence on the target latent variable but exhibit relatively low performance, thus offering critical insights for prioritizing managerial actions, resource allocation, or policy interventions [27].

In PLS-IPA, latent variables are placed into four quadrants based on the combination of their importance and performance. latent variables with high importance and high performance should be maintained, while latent variables with importance but low performance should be prioritized for improvement. Latent variables with low importance and performance require minimal attention, while latent variables with high performance but low importance may indicate overallocation of resources. Thus, PLS-IPA serves as a strategic tool that helps decision-makers accurately identify priority areas for intervention to optimize organizational outcomes [26].

**Fig. 2:**
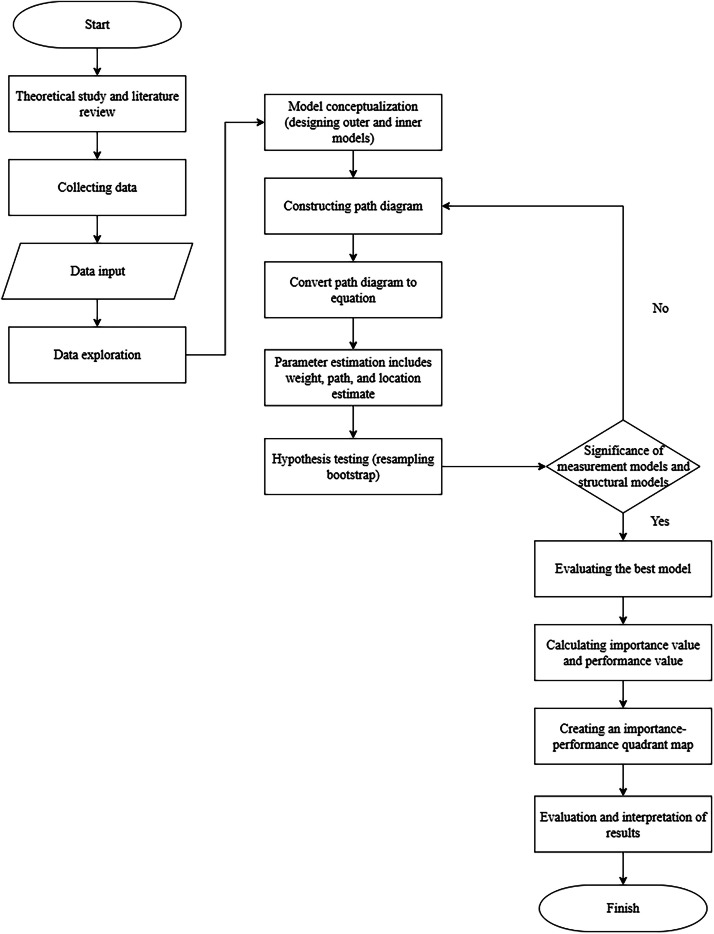
Research Flowchart.

The research methodology that has been described follows a sequential process outlined through the stages depicted in the flow diagram in Fig. 2.

To enhance the reproducibility of the proposed methodological approach, this study outlines a concise workflow for conducting PLS-IPA using SmartPLS 4. The procedure begins with specifying the structural and measurement models, ensuring that all latent constructs and indicators are appropriately defined. After data preparation and validation, the analysis proceeds to the IPMA module, which augments traditional PLS-SEM results by jointly evaluating construct importance and performance. Within the IPMA settings, the weighting scheme in the PLS setup is configured to PCA (Principal Component Analysis). This choice replaces conventional path or factor weighting procedures by generating latent variable scores based on the principal components of their indicators. Other parameters—such as standardized results and default initial weights—are retained to maintain comparability across replications. These settings operationalize the methodological contribution of integrating PCA-based weights into the PLS framework.

The IPMA estimation is then executed by selecting *Start calculation*, prompting SmartPLS to compute both importance values (total effects on the target construct) and performance scores (latent variable means scaled from 0 to 100). The resulting report includes tabular and graphical outputs that help identify indicators or constructs with high importance but low performance, supporting targeted policy recommendations. To further strengthen reproducibility, researchers are encouraged to document the exact software version used, preserve the SmartPLS project file, and provide supplementary materials such as pseudocode or workflow summaries. Such documentation is particularly beneficial for users unfamiliar with PCA-based weighting schemes and ensures that the proposed PCA-enhanced PLS-IPA procedure can be implemented consistently across future studies [22].

## Method validation

### Data collection

This study uses secondary data sourced from the 2022 East Java Health Profile, the 2022 East Java Education Statistics, the 2022 Food Security Index Book, and several publications from Badan Pusat Statistik (BPS) in 2022. The data represent conditions in 2022 with the analysis unit covering 38 districts/cities in East Java Province. The study focused on five latent variables, namely socioeconomic status, food security, childcare practices, health and environmental services, and child malnutrition status. Each latent variable is measured through a number ofindicators compiled based on the conceptual framework and references from previous studies [[28], [29], [30], [31], [32], [33]]. Details of the latent variables and their indicators are shown in Table 1.

**Table 1:** Research variable.

Latent Variable	Indicator	
Socio-Economic	X_1.1_	Per capita expenditure
	X_1.2_	Average years if schooling
	X_1.3_	Percentage of poor population
Parenting	Y_1.1_	Proportion of infants exclusively breastfed
Food Security	Y_2.1_	Food security index score
Health and Environmental Services	Y_3.1_	Proportion of toddlers receiving vitamin A
	Y_3.2_	Proportion of pregnant women receiving iron tablets
	Y_3.3_	Proportion of toddlers fully immunized
	Y_3.4_	Proportion of neonatal visits for infants
	Y_3.5_	Percentage of households with access to safe drinking water
	Y_3.6_	Percentage of households with access to proper sanitation
	Y_3.7_	Coverage of antenatal care for pregnant women
Malnutrition Status	Y_4.1_	Prevalence of stunting
	Y_4.2_	Prevalence of wasting
	Y_4.3_	Prevalence of underweight

### Parameter estimation

The parameter estimation in the PLS-SEM modeling in this study used the PCA scheme. The PCA scheme was chosen because it can generate latent variable scores based on linear combinations of its indicators, thus capturing the greatest variance in the data. The PCA scheme also supports the research objective of identifying relationships between latent variables in the model of malnutrition in toddlers in East Java.

Parameter estimation was performed using the least squares method to obtain the parameter coefficients for the measurement model and the parameter coefficients for the structural model. The parameter coefficients for the measurement model are as follows. a. Exogenous Latent Variable Indicators

$${\hat{\lambda }}_{x_{1.1}}=0,966\;{\hat{\lambda }}_{x_{1.2}}=0,966$$

b. Endogenous Latent Variable Indicators

**Table:** 

${\hat{\lambda }}_{y_{1.1}}=1,000$	${\hat{\lambda }}_{y_{2.1}}=1,000$${\hat{\lambda }}_{y_{4.3}}=0,975$	${\hat{\lambda }}_{y_{3.1}}=0,639$ ${\hat{\lambda }}_{y_{3.2}}=0,469$ ${\hat{\lambda }}_{y_{3.3}}=0,779$ ${\hat{\lambda }}_{y_{3.4}}=0,192$ ${\hat{\lambda }}_{y_{3.5}}=0,052$ ${\hat{\lambda }}_{y_{3.6}}=0,411$ ${\hat{\lambda }}_{y_{3.7}}=0,765$	${\hat{\lambda }}_{y_{4.1}}=0,892$ ${\hat{\lambda }}_{y_{4.2}}=0,879$

The coefficient values of the structural model parameters are as follows:

**Table:** 

${\hat{\gamma }}_{11}=0,464$ ${\hat{\gamma }}_{21}=0,720$ ${\hat{\gamma }}_{31}=0,513$	${\hat{\beta }}_{41}=-0,185$ ${\hat{\beta }}_{42}=0,178$ ${\hat{\beta }}_{43}=-0,011$

These values are then entered into the equation so that the equation for each PLS scheme is as follows:

$$\left[\begin{array}{c} \eta_{1}\\ \eta_{2}\\ \eta_{3}\\ \eta_{4} \end{array}\right]=\left[\begin{array}{cccc} 0 & 0 & 0 & 0\\ 0 & 0 & 0 & 0\\ 0 & 0 & 0 & 0\\ -0,185 & 0,178 & -0,011 & 0 \end{array}\right]\left[\begin{array}{c} \eta_{1}\\ \eta_{2}\\ \eta_{3}\\ \eta_{4} \end{array}\right]+\left[\begin{array}{cccc} 0,464 & 0 & 0 & 0\\ 0,720 & 0 & 0 & 0\\ 0,513 & 0 & 0 & 0\\ 0 & 0 & 0 & 0 \end{array}\right]\left[\begin{array}{c} \xi_{1}\\ \xi_{2}\\ \xi_{3}\\ \xi_{4} \end{array}\right]+\left[\begin{array}{c} \zeta_{1}\\ \zeta_{2}\\ \zeta_{3}\\ \zeta_{4} \end{array}\right]$$

**Fig. 3:**
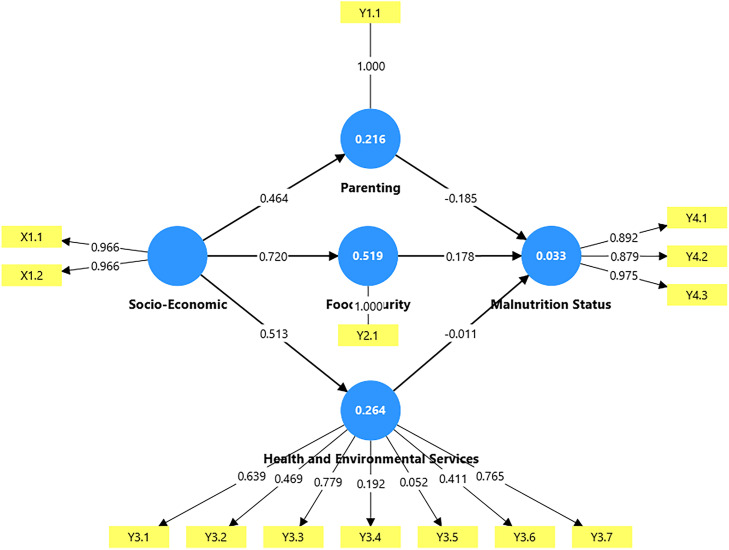
Research path diagram and parameter estimation.

The results of the parameter estimation obtained can also be seen in Fig. 3.

### Model evaluation

• Measurement Model Evaluation

As a first step, a measurement model evaluation was conducted to identify valid and reliable indicators for each latent variable. This process included testing the validity and reliability of the indicators in relation to their respective latent variables. Indicator validity was assessed through the factor loading value, which represents the level of correlation between the indicator and the latent variable it measures.

Validity evaluation was conducted by observing the loading factor value for each indicator. Indicators were declared valid if they had a loading factor > 0.5, while indicators with a loading factor < 0.5 were deemed invalid and therefore needed to be eliminated because they were unable to adequately represent the latent variable. Based on Table 2, several indicators had loading factors < 0.5: the proportion of pregnant women receiving iron supplements (Y_3.2_), the proportion of neonatal visits for infants (Y_3.4_), the percentage of households with access to safe drinking water (Y_3.5_), and the percentage of households with proper sanitation (Y_3.6_). Meanwhile, other indicators with loading factors > 0.5 were declared valid in reflecting the latent variables being measured. Table 2 shows that per capita expenditure and average years of schooling contributed equally to shaping the socioeconomic latent variable. In the health and environmental services latent variable, the proportion of toddlers receiving complete basic immunizations was recorded as the indicator with the largest contribution. Meanwhile, in the malnutrition status latent variable, the most dominant indicator was the prevalence of underweight.

**Table 2:** Evaluation of convergent validity with loading factor values.

Latent Variable	Indicator	Loadings	Information
Socio-Economic	X_1.1_	0.966	Valid
	X_1.2_	0.966	Valid
Parenting	Y_1.1_	1.000	Valid
Food Security	Y_2.1_	1.000	Valid
Health and Environmental Services	Y_3.1_	0.639	Valid
	Y_3.2_	0.469	Not valid
	Y_3.3_	0.779	Valid
	Y_3.4_	0.192	Not valid
	Y_3.5_	0.052	Not valid
	Y_3.6_	0.411	Not valid
	Y_3.7_	0.765	Valid
Malnutrition Status	Y_4.1_	0.892	Valid
	Y_4.2_	0.879	Valid
	Y_4.3_	0.975	Valid

The convergent validity test was also continued by assessing the Average Variance Extracted (AVE). If the AVE value is > 0.5, the indicator is able to explain the variance of the latent variable well. The AVE values ​​for each latent variable can be seen in Table 3.

**Table 3:** Evaluation of convergent validity with AVE.

Latent Variable	AVE	Information
Socio-Economic	0933	Valid
Parenting	1000	Valid
Food Security	1000	Valid
Health and Environmental Services	0290	Not valid
Malnutrition Status	0840	Valid

Based on Table 3, it is known that the latent variables for health service facilities and the environment do not meet the convergence criteria. This finding is consistent with the results of previous tests based on factor loading values, which indicated that there were still invalid indicators within the latent variable.

Indicator validity was also tested using cross loading, which is used to assess discriminant validity. Cross loading indicates the level of correlation between an indicator and its original latent variable and other latent variables. Discriminant validity is considered good if the indicator's cross-loading value with the original latent variable is higher than its correlation with other latent variables. The cross loading values for the indicators are shown in Table 4.

**Table 4:** Evaluation of discriminant validity with cross loading values.

Latent Variable	Indicator	Latent Variable				
		Socio-Economic	Parenting	Food Security	Health and Environmental Services	Malnutrition Status
Socio-Economic	X_1.1_	**0.966**	0.438	0.639	0.490	−0.020
	X_1.2_	**0.966**	0.459	0.752	0.502	0.036
Parenting	Y_1.1_	0.464	**1.000**	0.499	0.401	−0.101
Food Security	Y_2.1_	0.720	0.499	**1.000**	0.498	0.081
Health and Environmental Services	Y_3.1_	0.170	0.455	0.179	**0.639**	0.059
	Y_3.2_	0.324	0.336	0.391	**0.469**	0.114
	Y_3.3_	0.177	0.312	0.298	**0.779**	0.132
	Y_3.4_	−0.069	−0.160	**−0.266**	0.192	−0.179
	Y_3.5_	**0.276**	0.127	0.173	0.052	0.195
	Y_3.6_	**0.590**	0.200	0.557	0.411	−0.188
	Y_3.7_	0.522	0.085	0.382	**0.765**	−0.112
Malnutrition Status	Y_4.1_	−0.101	−0.174	−0.032	−0.152	**0.892**
	Y_4.2_	0.099	−0.037	0.121	0.135	**0.879**
	Y_4.3_	0.024	−0.067	0.128	0.027	**0.975**

Table 4 shows that most indicators have higher cross-loading values for their latent variables compared to other latent variables, so it can be concluded that the measurement model has met discriminant validity well. However, there are still indicators with lower cross-loading values for their latent variables, namely the Proportion of neonatal visits to infants (Y_3.4_), the Percentage of households with access to clean drinking water (Y_3.5_), and the Percentage of households with proper sanitation (Y_3.6_). This finding confirms that both indicators are not valid in representing the measured latent variables.

Another method used to assess discriminant validity is the Fornell-Larcker criterion, which is the square root of the AVE value. A latent variable is said to have good discriminant validity if the square root of the AVE value is higher than the correlation between other latent variables. The results of testing using the Fornell-Larcker criterion are shown in Table 5. Based on Table 5, it can be seen that the Fornell-Larcker values for all latent variables are greater on the diagonal. This indicates that all indicators used have met validity requirements.

**Table 5:** Evaluation of discriminant validity with the Fornell-Larcker Criterion.

	Socio-Economic	Parenting	Food Security	Health and Environmental Services	Malnutrition Status
Socio-Economic	**0.966**	0.464	0.720	0.513	0.008
Parenting	0.464	**1.000**	0.499	0.401	−0.101
Food Security	0.720	0.499	**1.000**	0.498	0.081
Health and Environmental Services	0.513	0.401	0.498	**0.538**	0.004
Malnutrition Status	0.008	−0.101	0.081	0.004	**0.916**

Next, reliability testing was conducted to ensure the latent variables' internal consistency in measuring the latent variables. This reliability evaluation used composite reliability values as a measure. A latent variable is considered reliable if its composite reliability value exceeds 0.70. A summary of the composite reliability calculations for each latent variable is presented in Table 6.

**Table 6:** Reliability evaluation with composite reliability.

Latent Variable	Composite Reliability
Socio-Economic	0.965
Parenting	1.000
Food Security	1.000
Health and Environmental Services	0.687
Malnutrition Status	0.940

The reliability evaluation results in Table 6 are consistent with the validity test results, which indicate that the latent variables for health services and the environment do not meet the criteria. Meanwhile, the other latent variables have met reliability standards.

• Structural Model Evaluation

After the measurement model evaluation stage is complete, the next step is to evaluate the structural model to assess the relationships between latent variables. This process is carried out by reviewing the coefficient of determination (R²), Q-square predictive relevance, and goodness of fit (GOF) values. The R² values ​​for each endogenous latent variable in the model of malnutrition status among toddlers in East Java are shown in Table 7.

**Table 7:** Model determination coefficient value.

Endogenous Latent Variable	R²	Criteria
Parenting	0.216	Weak
Food Security	0.519	Moderate
Health and Environmental Services	0.264	Weak
Malnutrition Status	0.033	Weak

The R² value for the latent variable parenting patterns was 0.216, indicating that socioeconomic variables explained 21.6 % of the variation in parenting patterns, while the remaining 78.4 % was influenced by factors outside the model. The latent variable food security had an R² value of 0.519, meaning that 51.9 % of the variation was influenced by socioeconomic variables, while the remaining 48.1 % was influenced by external factors outside the model. For the latent variable health and environmental services, the R² value was 0.264, meaning that 26.4 % of the variation was explained by socioeconomic variables, while the remaining 73.6 % was influenced by other factors. Meanwhile, for the latent variable malnutrition status, food security, parenting patterns, and health and environmental services explained 3.3 % of the variation, with the remainder explained by factors outside the model.

The Q² value for predictive relevance was obtained through the following calculation.

$$\begin{array}{c} Q^{2}=1-\left(\left(1-R_{1}^{2}\right)\left(1-R_{2}^{2}\right)\left(1-R_{3}^{2}\right)\left(1-R_{4}^{2}\right)\right)\\ =1-\left(\left(1-0,216\right)\left(1-0,519\right)\left(1-0,264\right)\left(1-0,033\right)\right)\\ =0,732 \end{array}$$

The Q² value obtained indicates that the structural model has good predictive ability.

The overall model fit test was then performed by calculating the Goodness of Fit (GoF) value. The GoF value ranges from 0 to 1, with categories of 0.10 (low), 0.25 (moderate), and 0.36 (good). The GoF value calculation is shown below.

$$\begin{array}{c} GoF=\sqrt{\bar{AVE}\times \bar{R^{2}}}\\ =\sqrt{0,678\times 0,258}\\ =0,418 \end{array}$$

The calculation results show that the GoF value is 0.418, so it can be concluded that the model built is appropriate (fit) and has good ability to explain the data.

### Hypothesis testing

After evaluating and estimating the measurement and structural models, hypothesis testing is conducted based on the parameter estimation results of the measurement and structural models using *t*-test statistics. Hypothesis testing is used to demonstrate the level of significance of each parameter of each indicator in both the measurement and structural models. For the measurement model, the proposed hypothesis can be formulated as follows:

$H_{0}:\lambda_{i}=0$ (loading factor is not significant in measuring the latent variable)

$H_{1}:\lambda_{i}\neq 0$ (loading factor is significant in measuring the latent variable)

In this section, *i* represents the indicator index, with *i* *=* *1, 2, …, p*, where *p* indicates the total number of indicators. The significance of the measurement model parameters was evaluated using bootstrap resampling with the number of replications *B* = 2000 resamplings. The test statistic used was the *t*-test statistic according to Eq. (7). The decision to reject if the calculated t value > $T_{\left(\alpha ,df\right)}$ or p-value < α . The significance level used was α of 5 % and t-table = 1.96. Table 8 displays the t-statistics values in the measurement model hypothesis testing.

**Table 8:** Measurement model testing results with resampling bootstrap.

Latent Variable	Indicator	Loading Factor	T-Statistics	P-value	Information
Socio-Economic	X_1.1_	0.966	116.763	0.000	Valid, significant
	X_1.2_	0.966	116.763	0.000	Valid, significant
Parenting	Y_1.1_	1.000	*	*	
Food Security	Y_2.1_	1.000	*	*	
Health and Environmental Services	Y_3.1_	0.639	3.284	0.001	Valid, significant
	Y_3.2_	0.469	2.027	0.043	Valid, significant
	Y_3.3_	0.779	4.979	0.000	Valid, significant
	Y_3.4_	0.192	0.667	0.505	Not valid, not significant
	Y_3.5_	0.052	0.161	0.872	Not valid, not significant
	Y_3.6_	0.411	1.425	0.154	Not valid, not significant
	Y_3.7_	0.765	4.569	0.000	Valid, significant
Malnutrition Status	Y_4.1_	0.892	25.870	0.000	Valid, significant
	Y_4.2_	0.879	19.014	0.000	Valid, significant
	Y_4.3_	0.975	130.718	0.000	Valid, significant

Based on Table 8, hypothesis testing using the bootstrap resampling method supports the results of the validity and reliability evaluation, where there were still insignificant indicators in the latent variables of health and environmental services. In accordance with methodological guidelines, these invalid indicators, such as Y_3.4_, Y_3.5_, and Y_3.6_, were removed from the measurement model to enhance model robustness and improve the accuracy of construct measurement. Eliminating indicators with weak psychometric properties strengthens convergent validity, reduces measurement error, and yields more stable structural path estimates. By removing these indicators, the refined measurement model provides a more coherent and reliable representation of the latent variables [34]. Therefore, in the subsequent PLS-IPA analysis, only indicators that are both valid and statistically significant were included, ensuring that the importance-performance mapping is based on robust measurement properties and yields meaningful managerial or policy insights. Moreover, the reduction of the measurement model enhances the interpretability of the results by concentrating the analysis on indicators that genuinely represent the underlying construct. This refinement ensures that the subsequent policy recommendations derived from the PLS-IPA are more targeted, credible, and aligned with the most diagnostically relevant aspects of health and environmental services. The hypotheses tested in the structural model are formulated as follows.

1. The effect of socio-economic on parenting.

$$\begin{array}{c} H_{0}:\gamma_{11}=0\\ H_{1}:\gamma_{11}\neq 0 \end{array}$$

2. The effect of socio-economic on food security.

$$\begin{array}{c} H_{0}:\gamma_{21}=0\\ H_{1}:\gamma_{21}\neq 0 \end{array}$$

3. The effect of socio-economic on health and environmental services.

$$\begin{array}{c} H_{0}:\gamma_{31}=0\\ H_{1}:\gamma_{31}\neq 0 \end{array}$$

4. The effect of parenting on malnutrition status.

$$\begin{array}{c} H_{0}:\beta_{41}=0\\ H_{1}:\beta_{41}\neq 0 \end{array}$$

5. The effect of food security on malnutrition status.

$$\begin{array}{c} H_{0}:\beta_{42}=0\\ H_{1}:\beta_{42}\neq 0 \end{array}$$

6. The effect of health and environmental services on malnutrition status.

$$\begin{array}{c} H_{0}:\beta_{43}=0\\ H_{1}:\beta_{43}\neq 0 \end{array}$$

The significance of the structural model parameters was evaluated using bootstrap resampling with the number of replications *B* = 2000 resamplings. The test statistic used was the *t*-test statistic according to (8), (9). The decision to reject if the calculated t value > $T_{\left(\alpha ,df\right)}$ or p-value < α . The significance level used was α of 5 % and t-table = 1.96. Table 9 displays the t-statistics values in testing the structural model hypothesis.

**Table 9:** Structural model testing results with resampling bootstrap.

Path	Coefficient	T-Statistics	P-value
Socio-Economic $\left(\xi_{1}\right)$ → Parenting$\left(\eta_{1}\right)$	0.464	3.963	**0.000**
Socio-Economic $\left(\xi_{1}\right)$ → Food Security $\left(\eta_{2}\right)$	0.720	10.641	**0.000**
Socio-Economic $\left(\xi_{1}\right)$ → Health and Environmental Services $\left(\eta_{3}\right)$	0.513	2.103	**0.036**
Parenting $\left(\eta_{1}\right)$ → Malnutrition Status $\left(\eta_{4}\right)$	−0.185	0.851	0.395
Food Security $\left(\eta_{2}\right)$ → Malnutrition Status $\left(\eta_{4}\right)$	0.178	0.771	0.441
Health and Environmental Services $\left(\eta_{3}\right)$ → Malnutrition Status $\left(\eta_{4}\right)$	−0.011	0.053	0.958

Table 9 shows that food security, parenting patterns, and health and environmental services do not significantly influence cases of malnutrition among toddlers in East Java. The variables that do significantly influence food security are socioeconomic factors, parenting patterns, and health and environmental services.

### PLS-IPA analysis results

**Fig. 4:**
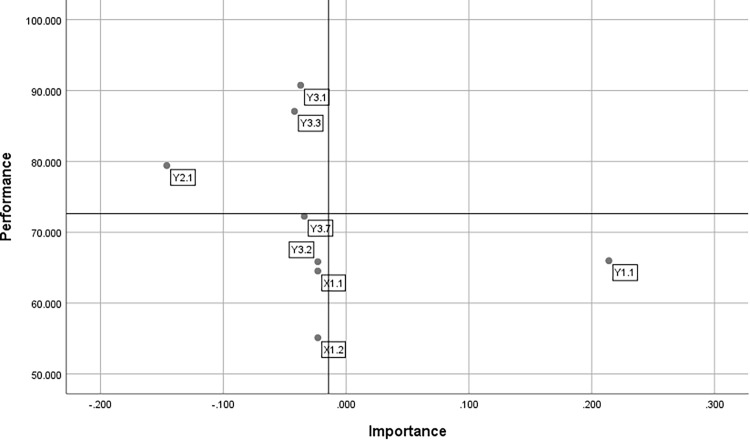
PLS-IPA Map.

After conducting the analysis using PLS-SEM, the indicators with significant influence were mapped in IPA. Fig. 4 is a map of the PLS-IPA results.

Based on Fig. 4, the following things can be interpreted.

• Top Priorities for Improvement

In the high importance but low performance quadrant, the indicator for the proportion of infants receiving exclusive breastfeeding (Y_1.1_) is found. This indicates that this indicator is considered important, but its performance remains low. Therefore, this indicator is a top priority for improvement through more targeted policy or program interventions to ensure optimal contribution.

• Low Priority

In the low importance and low performance quadrant, there are indicators such as per capita expenditure (X_1.1_), average years of schooling (X_1.2_), the proportion of pregnant women receiving iron tablets (Y_3.2_), and coverage of health services for pregnant women (Y_3.7_). These indicators have a low level of importance and their performance is also still weak. Therefore, these indicators are low priority and do not need to be a primary focus in the short term, although they still need to be considered in the long-term strategy.

• Excessive Potential

In the low-importance but high-performance quadrant, there are indicators such as food affordability score (Y_2.1_), proportion of children receiving vitamin A (Y_3.1_), and proportion of children receiving complete basic immunizations (Y_3.3_). These indicators demonstrate good performance, but their importance is relatively low. Therefore, there is potential for overallocation of resources to these indicators, necessitating re-evaluation to shift focus to more important indicators.

Based on the PLS-IPA results, several key findings can be interpreted in a structured manner. First, from the structural model, the path coefficients between latent variables show different patterns of influence: the Socio-Economic latent variable has a strong influence on Food Security ($\gamma_{21}$ = 0.720) and a moderate influence on Parenting ($\gamma_{11}$ = 0.464) and Health and Environmental Services ($\gamma_{31}$ = 0.513). The influence of Parenting on Malnutrition Status is negative and relatively small ($\beta_{41}$ = −0.185), while the influence of Food Security on Malnutrition Status is positive and relatively small ($\beta_{42}$ = 0.178), and the influence of Health and Environmental Services on Malnutrition Status is almost zero ($\beta_{43}$ = −0.011). The loading values in the measurement model also indicate the quality of the indicators: several indicators, such as X_1.1_ and X_1.2_, have very high loadings (≈0.966), as does indicator for the Malnutrition Status latent variable, which demonstrates measurement consistency (≈0.879–0.975). It should be noted that the (+/−) signs on the coefficients should be interpreted according to the scale and direction of the variable coding; for example, a negative sign for the Parenting → Malnutrition Status relationship indicates an inverse relationship according to the variable definition in this study. The combination of PLS results with IPA mapping enhances interpretation: IPA provides important information about the importance of each indicator to the target latent variable, along with its performance on the rescaled scale. In the resulting IPA map, indicator Y_1.1_ (proportion of infants receiving exclusive breastfeeding) is located in the high priority quadrant (high importance, low performance). This indicates that although Y_1.1_ significantly contributes to the target latent variable, its current achievement is low—making it a candidate for priority intervention. Conversely, indicators such as X_1.1_ (per capita expenditure), X_1.2_ (average years of schooling), Y_3.2_ (proportion of pregnant women receiving iron tablets), and Y_3.7_ (maternal health service coverage) fall into the low-performance and low-importance quadrant; these indicators are categorized as low priority for immediate action, although they will continue to be monitored in the long-term strategy. On the other hand, indicators that show excessive potential—that is, high performance but low importance (e.g., Y_2.1_ food affordability score, Y_3.1_ vitamin A coverage, Y_3.3_ complete immunization coverage)—suggest the potential for overallocation of resources to areas that are less decisive in their impact on malnutrition status; these results recommend reallocating or reassessing resource priorities toward indicators with greater importance but lower performance (e.g., Y_1.1_).

The use of the Combination of Partial Least Squares Structural Equation Modeling (PLS-SEM) method — Scheme of Principal Component Analysis (PCA) — with Importance–Performance Analysis (IPA) provides a comprehensive analytical framework. PLS-SEM estimates the structural relationships between latent variables and measurement quality, while IPA maps the total contribution of indicators (importance) and their actual achievements (performance), facilitating policy prioritization. The application of the PCA scheme in the context of SEM-PLS is an iterative method for extracting principal components one by one without having to calculate the full covariance matrix. Its advantages include computational efficiency on large datasets, the ability to handle non-normally distributed data, and ease of integration with weight-based PLS estimation procedures. In SEM-PLS practice, PCA is used to latent variable composite scores or higher-order latent variables, then these weights are incorporated into the measurement and structural models to obtain consistent parameter estimates. The application of PLS-IPA enhances the analytical capabilities of research: in addition to assessing the direction and magnitude of influence between latent variables (through path coefficients), this method allows mapping of the most decisive but underperforming indicators so that policy recommendations can be more targeted and effective. By combining quantitative evidence from PLS (the magnitude and sign of coefficients, and the reliability of indicators through loading) and the results of IPA mapping (prioritization of actions based on importance vs. performance), program planners or policymakers gain dual insights—namely, where interventions will have the greatest impact and what needs to be improved first.

## Limitations

This study has several limitations. First, the analysis uses only aggregate data from 2022, making it cross-sectional and unable to capture the dynamics of change over time. Second, the data used are sourced from publications by the Central Bureau of Statistics, so their quality and completeness depend on the design and implementation of the official survey. Third, although the conceptual framework refers to the UNICEF 2020 model, this study only covers indirect and underlying causal factors, while direct causal factors such as dietary intake and infectious diseases could not be analyzed due to data limitations. Fourth, the PLS-SEM approach used is exploratory, so the relationships between variables represent associations rather than definitive causal relationships. Fifth, the integration of Importance–Performance Analysis (IPA) does provide a strategic overview, but it does not fully reflect contextual factors such as culture, local policies, and social dynamics that influence children's nutritional status.

## Ethics statements

The data used in this research are secondary data collected from official publications of East Java Province in 2022. The data are publicly available upon request from Badan Pusat Statistik (BPS), Indonesia.

## Supplementary material *and/or* additional information [Optional]

*None*

## CRediT authorship contribution statement

**Bambang Widjanarko Otok:** Conceptualization, Methodology, Validation, Supervision, Writing – review & editing. **Zulfani Alfasanah:** Validation, Visualization, Conceptualization, Methodology, Writing – original draft. **Diaz Fitra Aksioma:** Writing – review & editing.

## Declaration of competing interest

The authors declare that they have no known competing financial interests or personal relationships that could have appeared to influence the work reported in this paper.

## Data Availability

I have shared the link to my data at the attach file step
